# Molecular characterization of human *Echinococcus* isolates and the first report of *E. canadensis* (G6/G7) and *E. multilocularis* from the Punjab Province of Pakistan using sequence analysis

**DOI:** 10.1186/s12879-020-04989-6

**Published:** 2020-04-03

**Authors:** Aisha Khan, Haroon Ahmed, Sami Simsek, Hua Liu, Jianhai Yin, Ying Wang, Yujuan Shen, Jianping Cao

**Affiliations:** 1 Department of Biosciences, COMSATS University Islamabad (CUI), Islamabad, Pakistan; 2 Key Laboratory of Parasite and Vector Biology, MOH, Shanghai, China; 3 WHO Collaborating Centre for Tropical Diseases, Shanghai, China; 4 National Institute of Parasitic Diseases, Chinese Center for Disease Control and Prevention, Shanghai, China; 5 Department of Parasitology, Faculty of Veterinary Medicine, University of Firat, 23119 Elazig, Turkey

**Keywords:** *E. granulosus* sensu stricto, *E. canadensis*, *E. multilocularis*, Human, Genotyping, Pakistan

## Abstract

**Background:**

Echinococcosis is a zoonotic parasitic disease causing serious health problems in both humans and animals in different endemic regions across the world. There are two different forms of human echinococcosis: Cystic Echinococcosis (CE) and Alveolar Echinococcosis (AE). CE is caused by the larval stage of *Echinococcus granulosus* sensu *lato* and AE by the larval stage of *Echinococcus multilocularis*. Geographically, CE is universally distributed, while AE is prevalent in the northern hemisphere. Although the disease is endemic in neighboring countries (China, Iran and India) of Pakistan, there are limited reports from that country. Besides, there are no comprehensive data on the genotyping of *Echinococcus* species in humans based on sequence analysis. This study aimed to detect the presence of human CE and to identify *Echinococcus* spp. in human isolates through genetic characterization of hydatid cysts in the Punjab Province of Pakistan.

**Methods:**

Genetic analysis was performed on 38 human hydatid cyst samples collected from patients with echinococcosis using mitochondrial cytochrome c oxidase subunit 1 (*cox*1), cytochrome b (*cytb*) and NADH subunit 1 (*nad*1). Patient data including age, epidemiological history, sex, and location were obtained from hospital records.

**Results:**

According to the sequence analysis we detected *E. granulosus* sensu stricto (*n* = 35), *E. canadensis* (G6/G7) (*n* = 2), and *E. multilocularis* (*n* = 1). Thus, the majority of the patients (92.1%, 35/38) were infected with *E. granulosus s.s.* This is the first molecular confirmation of *E. canadensis* (G6/G7) and *E. multilocularis* in human subjects from Pakistan.

**Conclusions:**

These findings suggested that *E. granulosus s.s.* is the dominant species in humans in Pakistan. In addition, *E. canadensis* (G6/G7) and *E. multilocularis* are circulating in the country. Further studies are required to explore the genetic diversity in both humans and livestock.

## Background

Echinococcosis is a zoonotic disease caused by tapeworm parasites belonging to the *Echinococcus* genus. There are two main types of echinococcosis: Cystic Echinococcosis (CE) caused by *Echinococcus granulosus sensu lato* and Alveolar Echinococcosis (AE) caused by *E. multilocularis*. Additionally, polycystic echinococcosis (caused by *E. vogeli* and *E. oligarthra*) also occurs predominantly in South America [1]. CE has a worldwide geographical distribution [2]. Echinococcosis disrupts the economies of many countries, affecting approximately 2–3 million people [3]. The estimated human burden of CE was below 1 million disability adjusted life years (DALYs), but may increase above this figure. Annual losses caused by CE might reach 20 million US dollars [4]. The disease has a prevalence of about 1/100,000 in developed countries and can reach 200/100,000 in rural populations having close contact with domestic dogs [3].

Most species of *Echinococcus* inhabit domestic and wild mammals. The definitive hosts include both domesticated dogs and wild carnivore species (foxes, wolves and coyotes). Humans and livestock act as intermediate hosts. Livestock animals are the intermediate hosts for *E. granulosus s.s.*, while wild small mammals serve for *E. multilocularis*. Humans acquire infection with CE by accidental ingestion of parasite eggs in the contaminated food and water, or by direct interaction with the definitive hosts [5]. Hatching of eggs occurs in small intestine and then the developing parasite larvae can spread to any other organ; however, they prefer to reside in the liver, where parasite forms the hydatid cysts [6]. Molecular genotyping has shown that members of *E. granulosus s.l.* include *E. granulosus s.s.* (G1-G3), *E. equinus* (G4), *E. ortleppi* (G5), *E. canadensis* (G6/7, and G8–10) and *E. felidis* [2].

There are limited data about CE in Pakistan, whereas the incidence of the disease is high in neighboring countries such as Iran, India, and China, for which published data are available for both prevalence and genotyping. Limited research has been conducted on echinococcosis in the past decade in Pakistan [7]. Previous investigations of Pakistani isolates showed the incidence of *E. granulosus s.s.* (G1-G3) in cattle, buffalo and sheep, while in humans, *E. granulosus s.s*. (G1-G3) and *E. canadensis* (G6/7) were detected based on data using the *cox1* gene sequences [8–10]. However, interestingly, *E. multilocularis* was reported in cattle from Pakistan [10, 11]. In the past, *E. granulosus s.s.* was reported in livestock (e.g.,cattle), while *E. granulosus s.s.* and the former G6 genotype were reported by using polymerase chain reaction-restriction fragment length polymorphism (PCR-RFLP) analysis without confirmation by gene sequencing in humans in Khyber Pakhtunkhwa (KPK) province of Pakistan [11].

Therefore, in the current study, hydatid cyst samples collected from human patients with echinococcosis and stored as formalin-fixed paraffin-embedded (FFPE) tissues were subjected to sequence analysis by analyzing mitochondrial cytochrome *c* oxidase subunit 1 (*cox1*), cytochrome *b* (*cytb*), and NADH subunit 1 (*nad*1) genes, to investigate the possible genetic diversity in the hydatid cyst samples in Pakistan.

## Methods

### Geography of the study area

Punjab is one of the largest provinces by population, with fertile agricultural land and deserts in the southern part near the border with Rajasthan and near the Sulaiman Range. Punjab comprises parts of the Cholistan and Thal deserts. It has extreme weather, with foggy and wet winters. The average temperature increases from mid-February, with springtime weather continuing until mid-April, when the summer heat sets in June and July are very hot months.

### Collection of samples

The formalin fixed and paraffin embedded hydatid cyst samples were collected from the Pathology Departments of the contributing hospitals from 2012 to 2017. All patients were confirmed as being infected with echinococcosis (CE, *n* = 37; AE, *n* = 1) by histopathological investigation after surgery (detection of Periodic Acid-Schiff (PAS)-positive laminated layers, and or protoscoleces and/or hooklets). Patient data, including age, sex, and epidemiological history were recorded.

### Molecular analysis

#### Genomic DNA extraction

The genomic DNA (gDNA) isolation was performed using individual Formalin-fixed paraffin-embedded (FFPE) tissues. Sections of 10–15 μm thickness were taken from each cyst by using astndard microtome (Leica SM2000 R Sliding Microtome,Wetzlar, Germany) with disposable DNA-RNA free blades. Equipment was autoclaved or sanitized before use. Paraffin was removed by incubation in 1 ml of xylene for 10 min at 37 °C. The supernatant was discarded after centrifugation at 12000×*g* for 5 min. Samples were rehydrated in descending ethanol concentrations; excess ethanol was evaporated at room temperature. A genomic DNA isolation kit (TRANS Easy Pure FFPE Tissue Genomic DNA Kit, Code: EE191–01; Transgen biotech, Beijing, China) was used to extract total gDNA according to the manufacturer’s protocol, with a few modifications. Briefly, the tissue samples were digested at 56 °C overnight in lysis buffer (400 μl), and then the gDNA was extracted. Sterile distilled water (100 μl) was used to resuspend the pellet. The gDNA samples were stored at − 20 °C until further use [12].

#### PCR amplification and sequencing

The mitochondrial genes (*cox1*, *nad1*, and *cytb*) were amplified from the isolated gDNA as previously described [12, 13]. Amplification of the *cox1* (446 bp) and *cytb* genes (580 bp) was carried out using a thermocycler with the following PCR conditions: denaturation was done at 94 °C for 30 s, annealing at 54 °C for 30 s and extension at 72 °C for 60 s, for 35 cycles. Amplification of the *nad1* gene (900 bp) was performed with the following PCR conditions: denaturation at 95 °C for 60 s, annealing was done at 50 °C for 50 s and extension at 72 °C for 70 s, for 30 cycles [14]. All PCR amplifications were performed with a negative control comprising sterile distilled water instead of the DNA template. The PCR products were visualized using a gel doc system after separation through a 1.5% agarose gel. All positive samples PCR products were subjected to sequence analysis.

#### Phylogenetic analysis

Construction of the phylogenetic tree, multiple sequence alignment, and unidirectional DNA sequence analysis were constructed using Mega X [15]. A maximum composite likelihood (MCL) strategy was applied to construct the initial trees, using a heuristic search with the BioNJ algorithms and neighbor-joining approach. The superior log-likelihood value was applied to select the topology [70]. The reference sequences that were used as outgroups in the phylogeny and in tree construction are shown in Table 1.

**Table 1:** Provenance and GenBank accessions for the reference sequences used in the phylogenetic analyses

Species/Marker	*cox*1	*cytb*	*nad*1	Reference
*E. granulosus* (*s.s.*)	Portugal (HF947554)			[16]
	Argentina (MG672207) Algeria (MG672288) Greece (MG672282) Australia (MG672263) Mexico (MG672259) Tunisia (MG672171) Turkey (MG682535)	Algeria (MG672293) Iran (MG672246) Algeria (MG682544) Iran (MG682539) Spain (MG682527) Turkey (MG682536)	Algeria (MG672293) Greece (MG672282) Mexico (MG672259) Mongolia (MG672254) Finland (MG682511) India (MG682512) Albania (MG682514) Romania (MG682516) Turkey (MG682535) Italy (MG682521) Iran (MG682541) Turkey (MG682531)	[17]
	Greece (KU925430) Romania (KU925431) Spain (KU925419)			[18]
	Turkey (KY766888) Iran (KY766899) Morocco (EF367275) France (KY766892)		France (KY766893)	[19]
	Brazil (KT382540)^a^			–
	Argentina (KX039948)			[20]
	Palestine (KC109658)			[21]
*E. equinus*	Turkey (KY766905)	Turkey (KY766905)	Turkey (KY766905)	[19]
	UK (AB786665)	UK (AB786665)		[22]
			Germany (GQ420652)	[23]
*E. ortleppi*			India (KY766908)	[19]
			Japan (AB235846)	[2]
*E. canadensis (G6/G7)*	Kenya (KX010838)			[24]
		Mauritania (MH300954) Sudan (MH300948) Kenya (MH300938) Argentina (MH300934) Lithuania (MH301020) Ukraine (MH301022) France (MH300997)	Sudan (MH300939) Lithuani (MH301020)	[25]
	Mongolia (AB893260)			[26]
	Japan (AB235847)			[2]
	Peru (AB777925)			[27]
	Mongolia (AB271236)^a^			–
		China (MG597240)^a^		–
		Japan (AB745463)		[22]
*E. multilocularis*		China (KY290787, KY290785, MF370869, MF370870)^a^		–
		China (KT965467)		[28]
		Poland (KY205667, KY205676, KY205670)		[29]
		Canada (KC549999, KC550006)		[30]
		Slovakia (AB461397) Japan (AB461399, AB477009) France (AB461396) Kazakhstan (AB461398) Mongolia (AB461402)		[31]
		Canada (JF751035)		[32]
		Japan (AB018440)		

^a^GenBank (otherwise unpublished)

#### Statistical analysis

Data was analyzed for statistical analysis by using Fisher’s exact test.

## Results

In the present study, 38 human hydatid cyst samples were collected from surgically confirmed patients with echinococcosis, from different areas of Punjab, Pakistan. The average age of the patients with CE was 32.73 (ranging from 5 to 75 years). The demographic characteristics of infected cases are summarized in Table 2.

**Table 2:** Epidemiological and clinical information for 38 patients with echinococcosis

Parameter	Factor	*n* (%)
Sex	Male	22 (57.8)
	Female	16 (42.2)
Housing location	Urban	9 (23.7)
	Rural	29 (76.3)
Contact with stray dogs	Yes	23 (60.5)
	No	15 (39.5)
Cyst location	Liver	19 (50.0)
	Lung	8 (22.5)
	Spleen	1 (2.5)
	Right side of pelvis	1 (2.5)
	Right forearm	1 (2.5)
	Gall-bladder	1 (2.5)
	Kidney	2 (5.0)
	Extradural cyst	1 (2.5)
	Kidney	1 (2.5)
	Hypochondrium	1 (2.5)
	Hepatogastric omentum	1 (2.5)
	Orbital biopsy	1 (2.5)
Cyst size	Large	13 (34.3)
	Medium	05 (13.1)
	Small	20 (52.6)
Therapy with albendazole	Administered	36 (94.7)
	Not administered	02 (5.3)
Serology	Positive	13 (34.2)
	Negative	02 (5.3)
	Data not available	23 (60.5)

Among the 38 human echinococcosis samples analyzed, 22 were from males (57.8%) and 16 (42.2%) were from females and the differences were not statistically significant (*χ*^2^ = 1.89, *df* = 1, *P* > 0.05). A larger proportion (76.3%) of echinococcosis cases was reported from rural areas, which have closer contact or association with dogs compared with that in urban areas (23.7%). The liver (50%) was most affected organ, followed by the lungs (22.5%), and others (Table 2).

### Genetic characterization of *Echinococcus isolates*

PCR amplification of the *cox1* gene yielded a product of 446 bp, while *cytb* yielded a 580 bp fragment, and *nad1* yielded a 900 bp product in all samples. The nucleotide sequences of all Pakistani samples (*n* = 38) were BLAST searched against reference sequences retrieved from GenBank. According to the BLAST analysis of the sequences of the *cox1*, *cytb,* and *nad1* genes, *E. granulosus s.s.* (*n* = 35), *E. canadensis* (G6/G7) (*n* = 2), and *E. multilocularis* (*n* = 1) were detected. The findings showed that majority of the patients (35/38) were infected with *E. granulosus s.s*. All sequences have been published in GeneBank (acession no: MK229294–MK229342).

*E. granulosus s.s.* and *E. canadensis* (G6/G7) were characterized by using the sequences of *cox1* (446 bp), *cytb* (580 bp), and *nad1* (900 bp). Each sample was characterized by using the sequence at least one of them while *E. multilocularis* was identified by using only *cytb* (580 bp).

### Alignment results of the sequences

**Fig. 1:**
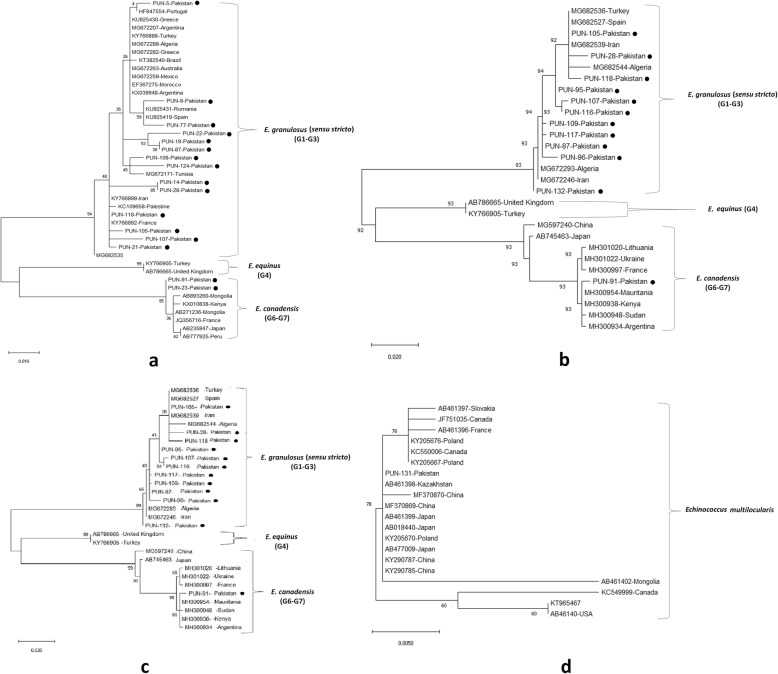
Comparison of the phylogenies of different genotypes within *Echinococcus* spp. based on *cox1* (446 bp) (**a**), *cytb* (580 bp) (**b**), *nad*1 (900 bp) (**c),** and (**d**) cytb (580 bp) only for *E. multilocularis.* Phylogenetic trees were constructed using neighbor-joining distance method analysis with a Tamura-Nei model [29]. The reliability of these trees was assessed using bootstrap analysis with 1000 replicates. Bootstrap support is shown at the nodes. In panel a, the clade of *E. equinus* (G4) and *E. canadensis* (G6-G7) can be seen clustering with *E. granulosus* (G1-G3) with very significant Bootstrap value of 95. In panel b, the cluster of (G4) and (G6-G7) is also further grouping with (G1-G3) with a bootstrap value of 92. In panel c, a bootstrap value of 95 occurred at the clustering node of *E. granulosus* with mini cluster of *E. equinus, E. ortleppi* and *E. canadensis.* In panel d, all the different species of *E. multilocularis* is successfully clustered together with a high bootstrap value of 76. The results are presented with the country of origin, the genotype, and GenBank accession number; the circles indicate the sequences of *E. granulosus s.l.* from the present study. The scale-bars indicate the number of substitutions per site

In the sequence comparison, the *cox1* gene showed 100% match with *E. granulosus s.s*. except for isolates PUN-23 and PUN-91 were identified as *E. canadensis* (G6/G7) (Fig. 1a).

The *cytb* gene sequences matched with the selected reference gene sequences of *E. granulosus s.s.* However, only PUN-91 was identical with the *E. canadensis* (G6/G7) reference gene sequence (Fig. 1b).

For the *nad1* gene, while the PUN-91 sequence matched with *E. canadensis* (G6/G7), all the other sequences were detected as *E. granulosus s.s.* after BLAST analysis (Fig. 1c).

Two samples were characterized as containing *E. canadensis* (G6/G7) based on *cox1* (446 bp). They were 100% identical and showed 100% similarity with a pig G6 isolate (GenBank: JQ356716) from France and 99.8% similarity with a human G6 isolate (GenBank: AB893260) from Mongolia (Fig. 1a). However, only one of them was identified as being *E. canadensis* (G6/G7) using the *nad1* and *cytb* sequences (Fig. 1b, c). The current study reveals the first report of genotype *E. canadensis* (G6/G7) in humans from the Punjab province in Pakistan (Table 3). The Pakistani isolate (PK-91) had 99.6% similarity with a G6 genotype (GenBank: MH300954) from Mauritania and with a human G6 genotype (GenBank: MH300938) from Kenya (Fig. 1c).

**Table 3:** Genotype assigned in relation to patient age, sex, and cyst localization

Parameter	No. of patients (%)	Genotype assigned
Age (years)		
1–10	3 (7.9)^a^	*E. granulosus s.s.*
11–20	6 (15.8)^a^	*E. granulosus s.s.*; *E. canadensis* (G6/G7)
21–30	12 (31.6)^b^	*E. granulosus s.s.*
31–40	4 (10.5)^a^	*E. granulosus s.s.*; *E. multilocularis*
41–50	7 (18.4)^a^	*E. granulosus s.s.*
51–60	4 (10.5)^a^	*E. granulosus s.s.*
> 60 or 71–80	2 (5.3)^a^	*E. granulosus s.s.*
Sex		
Male	22 (57.8)^c^	*E. granulosus s.s.*; *E. canadensis* (G6/7); *E. multilocularis*
Female	16 (42.2)^c^	*E. granulosus s.s.*
Cyst location		
Liver	19 (50.0)^d^	*E. granulosus s.s.*; *E. multilocularis*; *E. canadensis (G6/G7)*
Lung	8 (22.5)^e^	*E. granulosus s.s.*
Spleen	1 (2.5)^f^	*E. granulosus s.s.*
Right side of pelvis	1 (2.5)^f^	*E. granulosus s.s.*
Right forearm	1 (2.5)^f^	*E. granulosus s.s.*
Gall-bladder	1 (2.5)^f^	*E. granulosus s.s.*
Kidney	2 (5.0)^f^	*E. granulosus s.s.; E. canadensis (G6/G7)*
Extradural cyst	1 (2.5)^f^	*E. granulosus s.s.*
Kidney	1 (2.5)^f^	*E. granulosus s.s.*
Hypochondrium	1 (2.5)^f^	*E. granulosus s.s.*
Hepatogastric omentum	1 (2.5)^f^	*E. granulosus s.s.*
Orbital biopsy	1 (2.5)^f^	*E. granulosus s.s.*

^a,b^ Age: The different letters indicate statistically significant differences (*χ*^2^ = 12.71, *df* = 6, *P* = 0.41)

^c^ Sex: No statistically significant difference between sexes (*χ*^2^ = 1.89, *df* = 1, *P* = 0.25)

^d,e,f^ Cyst location: The different letters indicate statistically significant differences (*χ*^2^ = 61.89, *df* = 11, *P* = 0.00)

One *cytb* gene sequence (580 bp) was identified as *E. multilocularis* and it was 100% identical to isolates from China (GenBank: KY290787 and KY290785), 99.8% identical to isolates from foxes in Poland (GenBank: KY205676 and KY205667), and 99.6% identical to an isolate from a fox in France (GenBank: AB461396). Meanwhile, the Pakistani strains were genetically distant from the Mongolian and Canadian strains, as shown in the phylogenetic tree (Fig. 1d).

**Fig. 2:**
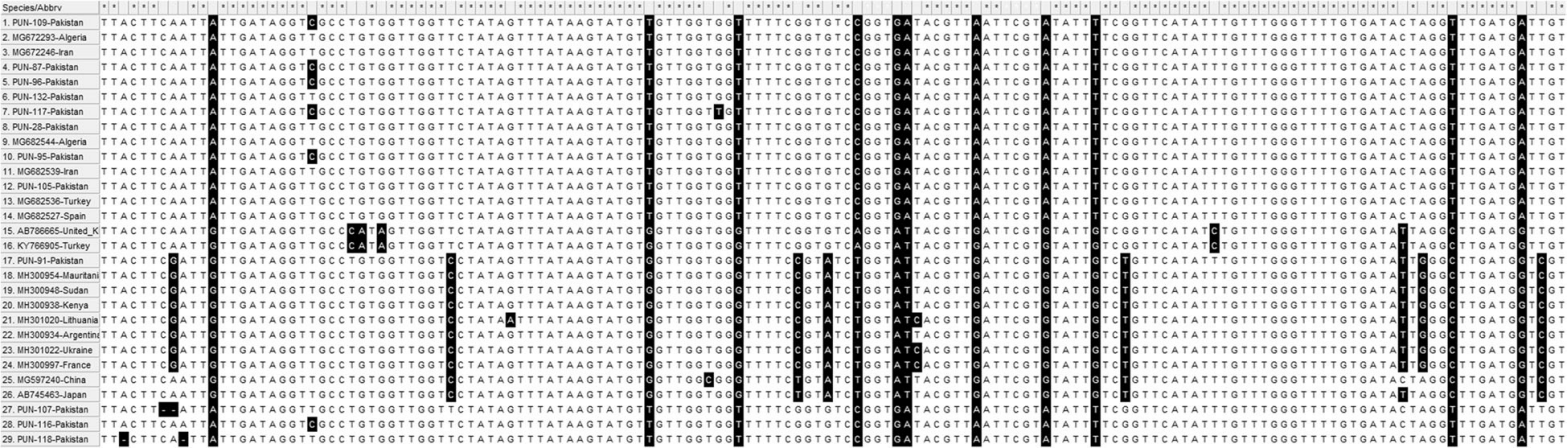
Multiple sequence alignments of partial *cytb* gene sequences. Genotypes (G1, G3, G5 and G6), represented with PUN suffixes, were from this study, while reference sequences from GenBank of genotypes G1, G3, G5 and G6 are presented with different codes. The accession numbers range from MK229294 to MK229342

The *cytb* gene from the Pakistan samples of *E. granulosus* (*s.s.*) showed 100% similarity with the other selected reference sequences. All other compared G6, G7, and G10 samples, including the samples from Pakistan, showed the same sequences (Fig. 2).

**Fig. 3:**
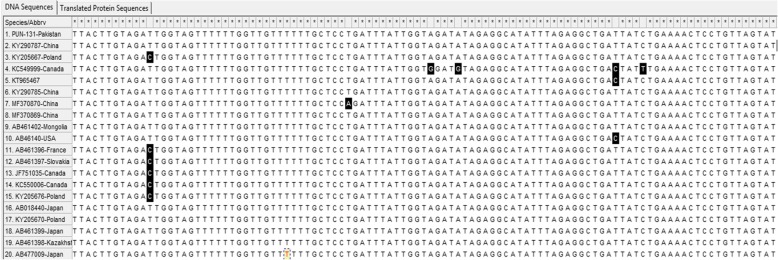
Multiple sequence alignments of partial *cytb* gene sequences (*E. multilocularis*). Genotypes (G1, G3, G5 and G6) represented with PUN suffix were from this study while reference sequences from GenBank of genotypes G1, G3, G5 and G6 are presented with different codes. The accession numbers range from MK229294 to MK229342

**Fig. 4:**
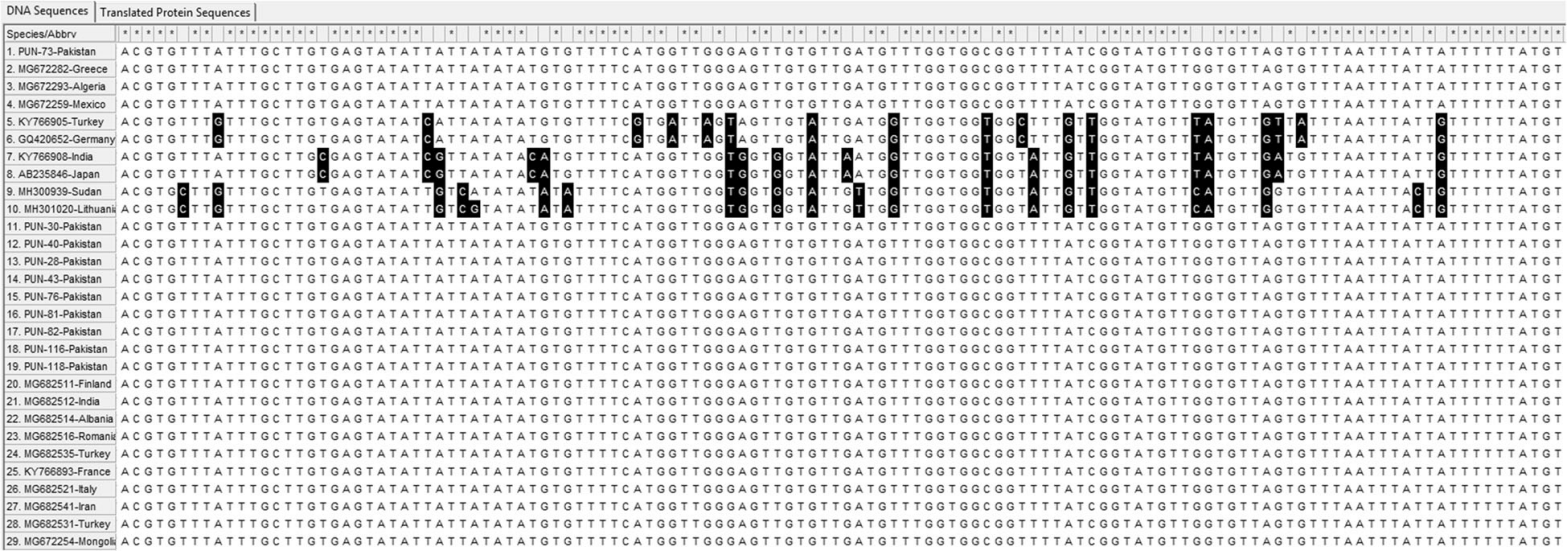
Multiple sequence alignments of partial *nad*1 gene sequences. Genotypes (G1, G3, G5 and G6), represented with PUN suffixes, were from this study, while reference sequences from GenBank of genotypes G1, G3, G5 and G6 are presented with different codes. The accession numbers range from MK229294 to MK229342

For the *nad1* gene, PUN-131-Pakistan was conserved when compared with the selected genotypes, whereas point mutations and substitutions were found in some of the other compared sequences (Fig. 3). The PUN-116-Pakistan sample had point mutations reported from France (GenBank: KY766893) and Turkey, while other sequences of *E. granulosus* (*s.s.*) were conserved (Fig. 4).

## Discussion

The two notable cestode-borne zoonoses are CE and AE. In the northern hemisphere, AE is widely distributed, while CE is widely distributed across the world and the disease burden in humans is highly variable in different endemic areas. AE and CE are still considered as neglected zoonoses in many areas of the world, although their prevalence is quite high in such areas, because of lack of awareness and disease management. The occurrence of CE is quite high around the world; however, the pathogenicity and fatality caused by AE is more prevalent in Asia [33]. CE is an endemic disease in Pakistan and causes serious economic losses in terms of human healthcare and livestock agriculture costs. In addition, there is lack of knowledge about CE in Pakistan that affects its transmission dynamics [34–36]. Agriculture is the backbone of the Pakistan and a large number of families are affiliated with this sector, including animal rearing and dairy farming for milk products. In small and domestic farms, standard principles are often not strictly followed; therefore, these populations are at high risk of acquiring *Echinococcus* spp*.* infection [7]. CE is considered a socially constructed disease because of various traditional practices found among different ethnic groups around the globe, such as keeping many dogs and a large amount of livestock, and the culture of rescuing stray dogs [37].

In current investigation, a total of 35 hydatid cyst samples were characterized as resulting from *E. granulosus s.s.* A high rate of *E. granulosus s.s.* was detected, which is in line with the data reported previously in humans (88.5%) [38] and livestock [39]. Similarly, in China, the majority (60%) of CE positive cases in humans are caused by *E. granulosus s.s.* (formerly the G1 strain) [40], which also caused 40.62% of the infections reported in India [41]. However, there is little information on the genetic characterization of *Echinococcus* spp*.* in humans in Pakistan. *Echinococcus granulosus s.s.* has been reported in buffaloes in Sindh Province of Pakistan [8]. This species was detected in small and large ruminants, while the sheep strain (G1) was found in human samples (*n* = 2) using *cox1* gene sequencing [9]. *Echinococcus granulosus s.s.* in cattle has been reported in Pakistan [10]. The high rate of *E. granulosus s.s.* reported in current study might be because *E. granulosus s.s.* is the predominant species in Pakistan (so far) and in neighbouring countries [9, 39, 40]. Even globally, *E. granulosus s.s.* is the most predominant causitive agent of CE [38]. It has a wide host range, which makes it more dominant in endemic localities even in cases where it occurs in sympatry with other *E. granulosus s.l.* In addition, it might reflect the fact that the maximum number of cases with CE were inhabiting in rural areas, where people have a close association with dogs [41].

In the present study, two samples were characterized as being infected with *E. canadensis* (G6/G7). *E. canadensis* (G6/7) was thought to be less infective to humans [42]. It is now known to be the second most important causative agent of CE after *E. granulosus s.s* [38]. Globally, *E. canadensis* (G6/7) has been reported in Kenya [42, 43]; Argentina [44]; China [40]; in different parts of Africa, Asia, and South America [12, 27, 45]; and in many countries in eastern and south-eastern Europe [27, 46, 47]. Meanwhile, the G6-G10 cluster was reported in Northern Palearctic, Northern Africa, and in the Middle East [27]. In Pakistan, because of the camel and pig populations, G6 transmission to human hosts is possible, especially resulting from camel slaughtering and cross boundary migration of animals from Afghanistan. The characterization of the *E. canadensis* (G6/G7) in humans in Pakistan suggests the interaction between the camel-dog and pig-dog cycles. In Pakistan, the pig population is abundant and imposes a serious health threat to the human population. Often, wild pigs live near human settlements in Pakistan. Although the camel population is quite low in the Punjab Province of Pakistan, sharing a border with Afghanistan and Iran means that species transmission is possible because of illegal animal transport across the border. Camels and other livestock animals live together; therefore, there is a possibility of exposure to other genotypes through interaction with the common definitive hosts, especially dogs. We could not compare our samples with Afghanistan isolates because there are no *E. granulosus* sequences from Afganistan deposited in GenBank.

In the present study, one sample was characterized as being infected with *E. multilocularis.* In North America, human cases of AE caused by *E. multilocularis* have been reported [48, 49]. AE is also prevalent in the northern hemisphere and even in the neighbouring country of Afghanistan [33]. In Pakistan echinococoosis ingeglected yet [50, 51]. The current study is first report of genotyping of *E. multilocularis* from humans in Punjab Province, Pakistan using sequence analysis. Previously, *E. multilocularis* was investigated in cattle from the KPK province of Pakistan as assessed using PCR-RFP [11]. The present findings suggest that cystic echinococcosis is an important emerging health issue and that AE is circulating in rural areas of Pakistan.

## Conclusions

In conclusion, the current findings indicate the presence of *E. granulosus s.s.*, *E. canadensis* (G6/G7), and *E. multilocularis* in the Punjab province of Pakistan. Additionally, *E. canadensis* (G6/G7) in human isolates is reported for the first time in Pakistan. To aid the eradication of the disease, comprehensive surveillance should be initiated. Control measures developed based on surveillance results could help to slow down the spread of the disease. The probable occurrence of other *E. granulosus s.l.* species indicate that further epidemiological studies using more Echinococcus isolates from all intermediate hosts (e.g. human and others), as well as definitive hosts, should be performed in different climatic regions of Pakistan.

## Data Availability

The datasets supporting the conclusions of this article are included within the article. The data ia avaliable for *cytb, cox1 & nad*1 accession numbers ranged from MK229294 to MK229342 under https://www.ncbi.nlm.nih.gov/pmc/about/intro/.

## Abbreviations

CE: Cystic echinococcosis
AE: Alveolar echinococcosis
FFPE: Formalin-fixed paraffin-embedded tissues
PAS: Periodic acid-Schiff
